# Chicago Gynecological Society

**Published:** 1885-03

**Authors:** 

**Affiliations:** 2330 Indiana avenue


CHICAGO GYNECOLOGICAL SOCIETY.

*Friday evening, Feb. 20th, 1885. Regular Meeting.*—The President, Dr. H. P. Merriman, in the chair.

I. Report of Dr. Christian Fenger, on Professor Byford's "Two Cases of Mural Pregnancy."

II. Intra-mural Leio-myoma, exhibited by Dr. E. C. Dudley.

III. Functions of the Membranes in Labor, by Dr. Henry T. Byford.

IV. Two interesting Cases in Obstetrics,—Inaugural Thesis,—by Dr. Charles Caldwell.

I. Professor Christian Fenger presented a report of his anatomical investigation of Professor Byford's "Two Cases of Mural Pregnancy." (CHICAGO MEDICAL JOURNAL AND EXAMINER, Jan., 1885). The report appears in full in another column.

Professor Christian Fenger, after a careful microscopical and macroscopical examination, pronounced Case, No. I, *tubo-ovarian* pregnancy, Case, No. II, *tubo-abdominal pregnancy*.

On motion, Professor Fenger's report was accepted.

II. Professor W. W. Jaggard presented, for Professor E. C. Dudley, an intra-mural leio-myoma of the uterus. The fundus was more particularly involved; the cavity of the uterus was but slightly enlarged, and the *endometrium* was normal. The tumor weighed about fifteen pounds. Both tubes and ovaries were removed with the uterus. The ovaries had undergone extensive cystic degeneration.

The tumor was removed, by abdominal section in the median line, with supravaginal amputation of the vaginal portion of the uterus, both ovaries and tubes, on Friday, Feb. 20th, at Mercy Hospital. The pedicle was surrounded by *écraseur*, and rubber ligature, before amputation; subsequently, the pedicle was secured in Dawson's Clamp, and treated after the extra-peritoneal method. The peritoneum was carefully stitched around the lower angle of the incision and parietal peritoneum was, thus, united to visceral peritoneum.

III. Dr. Henry T. Byford then read a paper entitled, "Functions of the Membranes in Labor."

Dr. Byford discussed fairly, clearly, and forcibly the possibility, probability and utility of the preservation of the membranes from rupture until they dilate, or aid in dilating the vulvo-vaginal orifice, as well as the cervix.

Dr. Byford's paper appears in another column.

*Discussion.*—Professor James H. Etheridge thought the paper well-timed.

Professor W. W. Jaggard, while not prepared to accept the thesis of the paper as a universal proposition, thought the attention to the functions of the membranes, advised by Dr. Byford, would go far to prevent cervical, vaginal and perineal laceration in many cases.

Professor D. T. Nelson said the membranes, when adherent to the cervix, as pointed out by Professor DeLaskie Miller, delayed labor. Under these conditions, puncture of the membranes was indicated. In practice, he tried to find out whether or no, the membranes were adherent to the cervix. If adherent, he punctured the membranes; if free, he always waited for spontaneous rupture.

Dr. Philip Adolphus agreed with the author of the paper in his conclusions, except in case of hydrammios. Under such pathological conditions, it was frequently necessary to rupture the membranes, in order to secure uterine contractions.

Professor J. Suydam Knox was in the habit of aiding dilatation of the vaginal os with the finger. He was under the impression that the membranes were seldom operative in the dilatation of the vulvo-vaginal orifice.

Dr. Edmund J. Doering had followed Dr. Byford's suggestion during the past year, and believed the method had prevented perineal lacerations in many cases. Premature detachment of the placenta was a possible danger from delayed rupture of the bag of membranes.

Dr. Edward Warren Sawyer believed that the membranes should not be punctured until the *os uteri* was dilated to the extent, which the pelvis, in the concrete case, would permit. The function of the bag of waters, in labor, was the dilatation of the *os uteri.* He ruptured the amnion in one-half his cases. There was as great variation in the development of the uterine muscular tissue as in the development of the biceps. Retardation of rupture of the bag of membranes might lead to uterine inertia, or to premature detachment of the placenta. To follow Nature's suggestions was a beautiful theory; but Nature caused the membranes to be ruptured, in the majority of cases, before dilatation of the vaginal os. He was in the habit of aiding dilatation of the uterine os by the introduction of one or two fingers within the canal of the cervix; he also tried to peel off the membranes around the internal os, and was conscious that he had materially influenced, in a favorable direction, the process of parturition.

The periphery of the *os uteri* was less than the periphery of the vaginal orifice. It was inconceivable, under such physical conditions, that the bag of membranes, protruding through a smaller, could dilate a larger orifice.

Professor Charles Warrington Earle agreed with Dr. Sawyer that natural processes should be imitated, in the puncture of the bag of membranes, after complete dilatation of the *os uteri.*

Dr. H. P. Merriman thought Dr. Byford's paper contained excellent advice for the young practitioner. The thesis could not be accepted as a universal proposition. Dr. Sawyer and Professor Earle had pointed out the conditions, in which the application of the method was contraindicated.

Dr. Henry T. Byford, in conclusion, said that he had distinctly pointed out in his paper, that the retardation of rupture of the membranes was advisable only in perfectly physiological labors. The Fellows of the Society had called attention to pathological conditions, to which the method did not apply.

Dr. Sawyer had read a paper on "Occipito-posterior Positions," before the "American Gynecological Society," last October. A plausible explanation of the fact, that anterior rotation did not occur in Dr. Sawyer's thirty-nine cases, might be found in the premature rupture of the membranes.

IV. The inaugural thesis of Charles Caldwell, A. M. (Dartmouth), M. D. (Harvard), entitled "Two Interesting Cases in Obstetrics," was read by the Secretary.

Dr. Caldwell's two interesting cases were, (1) Ante-partum Hæmorrhage, caused by Accidental Separation of the Placenta; (2) Breech Presentation, with an Unusual Complication. The paper will appear *in extenso* in the CHICAGO MEDICAL JOURNAL AND EXAMINER.

*Discussion.*—Dr. Sawyer was surprised that a physician of the present day should disregard the opinion of Velpeau in the exhibition of ergot, when the cavity of the uterus was not empty, as Dr. Caldwell did, when he gave that remedy in his case of accidental hæmorrhage, from premature detachment of the placenta.

Dr. Henry T. Byford thought that the clinical observations of Schatz justified the exhibition of small doses of ergot, even when the uterus contained the fecundated ovum.

Dr. Charles Caldwell was then elected Fellow of the Society.

The Society adjourned to meet at the Palmer House, Friday evening, March 20th, 1885.

Professor Christian Fenger will read a paper—with demonstration of specimens—entitled "Chronic Peri-uterine Abscess."

W. W. JAGGARD, M. D., *Editor*,
2330 Indiana avenue.

February 21st, 1885.

